# The characterization of tumor microenvironment infiltration and the construction of predictive index based on cuproptosis-related gene in primary lung adenocarcinoma

**DOI:** 10.3389/fonc.2022.1011568

**Published:** 2022-11-25

**Authors:** Kun Li, Lei-Lei Wu, Hui Wang, Hao Cheng, Hui-Min Zhuo, Yun Hao, Zhi-Yuan Liu, Chong-Wu Li, Jia-Yi Qian, Zhi-Xin Li, Dong Xie, Chang Chen

**Affiliations:** ^1^ Department of Thoracic Surgery, Shanghai Pulmonary Hospital, School of Medicine, Tongji University, Shanghai, China; ^2^ School of Pharmacy, Naval Medical University, Shanghai, China; ^3^ School of Medicine and School of Life Science and Technology, Shanghai Tenth People’s Hospital of Tongji University, Tongji University, Shanghai, China

**Keywords:** cuproptosis-related gene, lung adenocarcinoma, prognosis, immunotherapy, targeted therapy

## Abstract

**Objective:**

We aimed to use the cancer genome atlas and gene expression omnibus databases to explore the characterization of tumor microenvironment (TME) infiltration and construct a predictive index of prognosis and treatment effect based on cuproptosis-related genes (CRGs) in primary lung adenocarcinoma (LUAD).

**Methods:**

We described the alterations of CRGs in 954 LUAD samples from genetic and transcriptional fields and evaluated their expression patterns from three independent datasets. We identified two distinct molecular subtypes and found that multi-layer CRG alterations were correlated with patient clinicopathological features, prognosis, and TME cell infiltrating characteristics. Then, a cuproptosis scoring system (CSS) for predicting the prognosis was constructed, and its predictive capability in LUAD patients was validated.

**Results:**

Two molecular subtypes of cuproptosis (Copper Genes cluster A and cluster B) in LUAD were identified. Copper Genes cluster B had better survival than those with Copper Genes cluster A (*p <*0.01). Besides, we found that the infiltration of activated CD4^+^ T cells, natural killer T cells, and neutrophils was stronger in cluster A than in cluster B. Then, we constructed a highly accurate CSS to predict the prognosis, targeted therapy effect, and immune response. Compared with the low-CSS subgroup, the mutations of the *TP53*, *MUC16*, and *TTN* genes were more common in the high-CSS subgroup, while the mutation of *TP53*, *TTN*, and *CSMD3* genes were more common in the low-CSS subgroup than in high-CSS subgroup. The low-score CSS group had an inferior survival than high-score CSS group (*p <*0.01). In addition, CSS presented good ability to predict the immune response (area under curve [AUC], 0.726). Moreover, AZD5363 and AZD8186 were the inhibitors of *AKT* and *PI3K*, respectively, and had lower IC50 and AUC in the low-score CSS group than it in the high-score CSS group.

**Conclusions:**

CRGs are associated with the development, TME, and prognosis of LUAD. Besides, a scoring system based on CRGs can predict the efficacy of targeted drugs and immune response. These findings may improve our understanding of CRGs in LUAD and pave a new path for the assessment of prognosis and the development of more effective targeted therapy and immunotherapy strategies.

## Introduction

Lung cancer is still the leading cause of malignancies-related death worldwide (1). Non-small cell lung cancer accounts for 80-85% of all lung cancers, the major part of which is the type of adenocarcinoma (2, 3). The prognosis of lung adenocarcinoma (LUAD) is not satisfactory in clinical practice (4, 5). In addition, many factors affect the survival of LUAD, such as combined stage, treatment modality, and tumor response heterogeneity (6–8). There are many reasons for the various prognosis of patients with the same combined stage or/and similar treatment, including a difference in clinicopathological characteristics, tumor heterogeneity, and tumor microenvironment (TME) (9–11). Therefore, it is important to describe the significance of TME in the progression, treatment, and prognosis of LUAD. Previous studies explored the association between TME and different approaches to programmed cell death in LUAD (12); however, the relationship between TME and cuproptosis in LUAD is still not clear.

Cuproptosis is an approach to programmed cell death dependent on copper ions, which was proved in a recent study (13). Copper is an essential cofactor for all organisms but is toxic if concentrations exceed thresholds maintained by evolutionarily conserved homeostatic mechanisms. However, the mechanism by which excess copper induces cell death is unknown. Recently, the Broad Institute has discovered a new mechanism that differs from known cell death: cuproptosis (13). Cuproptosis occurs through direct binding of copper to the fatty acylated components of the tricarboxylic acid cycle, resulting in the abnormal aggregation of fatty acylated proteins and loss of iron-sulfur cluster proteins, leading to proteotoxic stress leading to cell death. Copper ions are involved in cell death like iron ions, and the Broad Institute article suggests that drug inhibition of mitochondrial respiration may be a disease-fighting strategy; in addition, cancer expresses a large number of lipoylated mitochondrial proteins and is a highly respirator that utilizes copper. The killing of cancer cells by ionic metal carriers may become a new method of cancer treatment (13, 14). Recently, some researchers explored the significance of cuproptosis-related genes (CRGs) in immune infiltration and prognosis for melanoma and clear cell renal cell carcinoma (15, 16). They found that CRGs were likely to be prognostic indicators and provided potential therapy insights (15, 16). However, there was no related report to study cuproptosis in LUAD.

The treatment approach of LUAD included surgery, chemotherapy, radiotherapy, targeted therapy, and immune therapy (17). In clinical practice, targeted therapy and immune therapy play important roles in improving survival, especially in advanced-stage patients (17). Regrettably, the prognosis of some patients is not satisfactory, though those patients have received targeted therapy or immune therapy before, as the drug sensitivity is poor in those patients (7, 18). Therefore, it is urgent to find a predictive tool to perform the evaluation of the tumor response to drug and explore novel target to increase the sensitivity to therapy. The CRGs may provide key information about therapy and prognostic assessment according to the previous findings in other malignancies (12, 16). Thus, we aimed to use the cancer genome atlas (TCGA) and gene expression omnibus (GEO) databases to explore the characterization of TME infiltration and construct predictive index of prognosis and treatment effect based on CRGs in primary LUAD.

## Materials and methods

### Data collection

We obtained data of clinical data and sample information of LUAD patients from the TCGA database (https://portal.gdc.cancer.gov/) using the R package TCGAbiolinks (19). The LUAD transcriptome fragments per kilobase million (FPKM) data from TCGA were downloaded from the UCSC Xena browser (GDC hub: https://gdc.xenahubs.net), which contained 507 LUAD tissues and 59 normal lung tissues. For TCGA-LUAD data, in order to eliminate the error caused by the quantitative mRNA abundance of FPKM in multiple samples, we converted FPKM to transcripts per kilobase million (TPM) values for standardization (20). The GSE68465, GSE11969, GSE72094 data sets were downloaded from the Gene Expression Omnibus database (http://www.ncbi.nlm.nih.gov/geo) (21, 22). The data were uniformly pre-processed using the Robust Multichip Average algorithm for background correction, quantile normalization, and log2-transformation (23). The probes were converted into corresponding genes using the annotation information available from the gene platforms (GPL96-57554).

### Variance analysis and gene set variation analysis

In order to investigate the expression pattern of CRGs in the LUAD patients’ tumor tissues and adjacent nontumor tissues, as well as different clinical subgroups, we used Wilcoxon’s method to calculate differential genes in clinical subgroups of immune subtype, epidermal growth factor receptor (EGFR) mutation, age, gender, pathological combined stage, pathological nodal (pN) stage, pathological tumor (pT) stage, pathological metastasis stage, chemotherapy, echinoderm microtubule-associated protein-like 4- anaplastic lymphoma kinase (EML4-ALK) fusion, and pathological type (24). Age was a continuous variable, and six years old could be selected as the interval to change it into a categorical variable. Kruskal test was selected for multi-category clinical variables. To investigate the differences in CRGs in biological and function processes, GSVA was performed by R packages ‘GSVA’ (version 1.34.0) (25, 26). The gene sets ‘h.all.v7.5.1.symbols.gmt hallmark’ in the Molecular Signatures Database (MSigDB) was selected as the reference gene set and a *p-*value of <0.01 was considered the threshold (27).

### Somatic mutations and copy number alteration

Somatic mutation and copy number variation (CNV) data were downloaded from the TCGA database. The high-frequency mutations in CRGs were visualized using OncoPlot. Based on Affymetrix SNP6.0 array copy number segmented data, GISTIC2.0 was used to analyze the change of regions of CNV in different groups. Deep deletions (GISTIC2 value: −2) were defined as homozygous losses in comparison to shallow deletions (GISTIC2 value: −1), which resulted in heterozygous loss. CNA gains were defined as GISTIC2 value +1, while amplifications were defined as GISTIC2 value +2. The threshold was Q-value<0.25, and the confidence level was 0.90. We used the ‘maftools’ package to provide visualization of regions of copy number variation across high and low group samples (28). Chromosome locations of copper death genes were represented by chromosome ring diagrams. The mutation frequency was plotted using the ‘ggplot’ package. The genomic location information of CRGs was downloaded from Gencode.v29.annotation.

### Tumor mutation burden

We downloaded the somatic mutation file and calculated each patient’s tumor mutation burden (TMB) score. The influence of TMB on patient OS was evaluated by Kaplan–Meier analysis and compared between the high and low groups by t-test. Maftools R package was used.

### Survival analysis of CRGs

Based on the CRGs obtained above, a univariable Cox regression model was used to screen the DEGs associated with good prognosis (overall survival, OS; progression free interval, PFI), which was performed using the ‘survival’ package in R with *p*<0.05 as the threshold. The median was used as a cutoff value to separate high and low expression groups. Log-rank test was performed, and the Kaplan-Meier survival analysis was used for further survival analysis. 18 CRGs were obtained. The R package “survival” and “survminer” were employed for survival analysis and drawing.

Based on TCGA data, the expression matrix of CRGs was obtained and the interaction between CRGs was calculated. The correlation coefficient and P value were calculated by Spearman method. Based on the survival analysis results obtained above, Cytoscape was used to map the interaction network of CRGs.

### Consensus clustering analysis of CRGs

Unsupervised clustering of TCGA samples was performed using the ConsensusClusterPlus algorithm based on the expression level of CRGs to identify different copper modification patterns. A consensus clustering algorithm was applied to determine the number of clusters and the stability of the discovered clusters. Euclidean distance calculation of similarity measures between clusters and K-means of unsupervised clustering were used to estimate the number of CRGs clusters. The optimal number of clusters was determined by the cumulative distribution function (CDF) and the delta area and analyzed using the ConsensusClusterPlus R package with 1,000 repeats.

Principal component analysis (PCA) was done on the CRGs with the prcomp() R function and visualized using the Factoextra R package (version 1.0.7). Eigengene values of the first dimension of PCA (DIM1) were then visualized through a dot plot. We used the function fviz_contrib() to identify the genes that contributed more than average to the DIM1 axis. We used the ‘pheatmap’ package (version 1.0.12) to visualize the differences in CRGs and clinical characteristics of TCGA samples between cluster A and B.

### Differential expression analysis and enrichment analysis

Based on the above cluster classification model, the TCGA data set was divided into two groups, and the DEGs between the two groups were calculated using the ‘Limma’ (Linear Models for Microarray Data) package (24). The significance criterion of differential genes was set as *p*<0.05 (BH-corrected), and the absolute value of log fold change was greater than 0.18 (丨log2(1.2)丨). Gene ontology (GO) and Kyoto Encyclopedia of Genes and Genomes (KEGG) pathway enrichment analyses were performed and visualized by using the R package ‘clusterProfiler’ (version 3.12.0), with a strict cutoff of *p* < 0.05 and false discovery rate (FDR) of less than 0.05 (25). Among the genes obtained above, the genes with significantly good prognosis were screened by the same method as before. We used the ‘pheatmap’ package (version 1.0.12) to visualize the differences in CRGs and clinical characteristics of TCGA samples between DEG-cooper-cluster A and B.

### Construction and validation of cuproptosis scoring system

Based on the DEGs of the above two subtypes, the univariable Cox regression analysis and Kaplan-Meier survival analysis were performed to obtain 474 DEGs with a good prognosis. We constructed a scoring system to quantify individual cuproptosis patterns in LUAD patients. The cuproptosis scoring system (CSS) was established based on the cuproptosis subtype-related DEGs. CSS was developed using principal component analysis (PCA), and an overall score was obtained by calculating the principal component sum.

CSS=∑(PC1_i_+PC2_i_)

With the median CSS values as the cutoff value, it was divided into high and low CSS groups. Patients with CSS values lower than the median risk score were categorized into the low-risk group, whereas those with CSS values greater than the median risk score were placed in the high-risk group.

The CSS scores were evaluated in TCGA and three GEO validation sets (GSE11969, GSE72094, GSE68465) to verify the prognostic effect. The prognostic effect of CSS scoring was analyzed by multivariate regression based on clinical data. The forest diagram is drawn using the ‘forestplot’ function package. In order to analyze and verify the similarity of CRGs expression level between the GEO validation data and TCGA data, the average value of CRGs expression level in these data was calculated respectively. Pearson correlation was used to calculate the correlation of CRGs expression between different data. Correlation heat map was drawn using ‘corrplot’ function package, and then correlation coefficients and significant p-values are also shown.

### Hallmark pathways between high- and low-score CSS groups

Single sample Gene Set Enrichment Analysis (ssGSEA) scores of the hallmark pathways between high and low CSS score groups were calculated by the ‘GSVA Bioconductor’ package (version 3.10), using pathway definitions from Molecular Signatures Database (MSigDB) Hallmark gene sets collection developed by Broad Institute (http://www.gsea-msigdb.org/gsea/msigdb/collections.jsp) (26). The Wilcoxon test was used for comparison of data between two groups.

### The analysis of immune cell, TME, PD-1, and PD-L1 between high- and low-score CSS groups

We used the ESTIMATE algorithm to evaluate the TME scores of each individual. In addition, the 23 human immune cell infiltration levels of a single sample were calculated by ssGSEA. Furthermore, the levels of immune cell infiltration were also determined using the CIBERSORT algorithm (29). We also analyzed the correlations between the two subtypes of PD-1, PD-L1, and CTLA-4 expression.

### Cellular and molecular characteristics of immune subtypes

The six immune categories of pan-cancer (C1-C6) and immune-related molecular features were calculated by Thorsson et al. (30). By using an immunogenomic analysis of more than 1000 tumor samples from 33 cancer types, six immune subtypes were identified, including wound healing (C1); IFN-γdominant (C2); inflammatory (C3); lymphocyte depleted (C4); immunologically quiet (C5); and TGF-βdominant (C6). The definitions and characteristics of C1-C6 are as follows:

C1 (Wound Healing) had elevated expression of angiogenic genes, a high proliferation rate, and a Th2 cell bias to the adaptive immune infiltrate. C2 (IFN-γ Dominant) had the highest M1/M2 macrophage polarization, a strong CD8 signal and, together with C6, the greatest TCR diversity. C3 (Inflammatory) was defined by elevated Th17 and Th1 genes, low to moderate tumor cell proliferation, and, along with C5, lower levels of aneuploidy and overall somatic copy number alterations than the other subtypes. C4 (Lymphocyte Depleted) displayed a more prominent macrophage signature, with Th1 suppressed and a high M2 response. C5 (Immunologically Quiet) exhibited the lowest lymphocyte, and highest macrophage responses, dominated by M2 macrophages. IDH mutations were enriched in C5 over C4. C6 (TGF-β Dominant) displayed the highest TGF-β signature and a high lymphocytic infiltrate with an even distribution of Type I and Type II T cells.

### Drug susceptibility analysis and immune response assessment

To explore differences in the therapeutic effects of chemotherapeutic and targeted drugs in LUAD cell lines between the high and low CSS score groups, the data that support the findings of this study were downloaded from Genomics of Drug Sensitivity in Cancer (GDSC) database (www.cancerrxgene.org) and Cancer Cell Line Encyclopedia (CCLE) (portals.broadinstitute.org/ccle). For the above data, the CSS scores were calculated and the differences in drug sensitivity between high and low CSS groups were analyzed. IC50 was quantified *via* the ‘pRRophetic’ package of R. Pearson method was used for correlation between CSS scores and IC50 of different drugs analysis.

We predicted the response to immune checkpoint blockade (ICB) through the TIDE website (http://tide.dfci.harvard.edu/login/). With the use of the Wilcoxon test, the difference in TIDE score between high and low CSS groups was compared. The predictive AUC of the CSS score was also calculated using TIDE scores.

### Statistical analyses

The data analyses and visualization were conducted in R (version 4.1.1), *and the following packages were used: “GEOquery,” “dplyr,” “Summarized Experiment,” “TCGA biolinks,” “readr,” “stringr,” “edgeR,” “org.Hs.eg.db,” “affy,” “limma,” “data.table,” “dplyr,” “zFPKM,” “ComplexHeatmap,” “ggplot2,” “ggalt,” “ggpubr,” “maftools,” “ggthemes,” “ggsignif,” “reshape,” “tidyverse,” “viridis,” “gridExtra,” “hrbrthemes,” “ggstatsplot,” “RColorBrewer,” “ggsci,” “RCircos,” “rtracklayer, rgl,” “pca3d,” “Rtsne,” “survival,” “survminer,” “ConsensusClusterPlus,” “RColorBrewer,” “pheatmap,” “GSVA,” “ggalluvial,” “plyr,” “forestplot,” “GSEABase,” “qusage,” “ggforce,” “survivalROC,” “ggsci,” “scales,” “maftools,” “enrichplot,” “gridExtra,” “TCGAbiolinks.”* The Wilcoxon test was used for comparison of data between two groups, whereas the Kruskal-Wallis test was for comparison of data among three groups. Univariate survival analysis was performed by K–M survival analysis with the log-rank test. Pearson method was used for correlation analysis. The tumor mutation burden (TMB) score was calculated by the package “maftools” in R. A p value <0.05 was considered statistically significant (* p < 0.05; ** p < 0.01; *** p < 0.001; **** p < 0.0001).

## Results

### Landscape of cuproptosis related genes in LUAD

**Figure 1:**
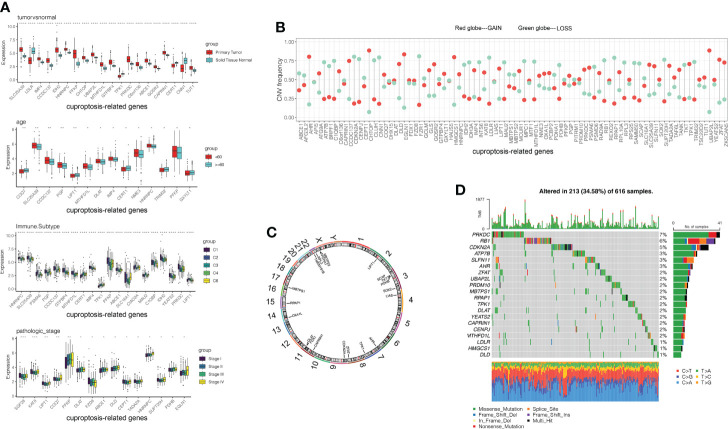
Genetic landscape and expression levels of CRGs in LUAD. **(A)** Expression of CRGs in clinical samples classified by tumor tissue, age, immune subtypes and pathological tumor stage. **(B)** Frequencies of CNV gain, loss, and non-CNV among 86 CRGs. **(C)** Locations of CNV alterations in CRGs on 23 chromosomes. **(D)** Mutation frequencies of CRGs in 507 patients with LUAD from the TCGA cohort. CGRs, cuproptosis-related genes; LUAD, lung adenocarcinoma; TCGA, The Cancer Genome Atlas; CNV, copy number variant.

After differential expression analysis in the TCGA dataset between 512 tumor tissues and 59 normal tissues, upregulated and downregulated DEGs were obtained. The 100 CRGs, including 63 upregulated genes and 37 down-regulated genes, listed in **Supplementary Table 1** were obtained from *Tsvetkov’s* study (13). After the intersection of the two groups of data, 86 CRGs remained. Based on TCGA gene expression data and clinical data, we further analyzed the expression differences of CRGs in LUAD patients’ tumor tissues and adjacent nontumor tissues, as well as differential genes expression levels in clinical classification samples, including immune subtype, epidermal growth factor receptor (EGFR) mutation, age, gender, pathological combined stage, pathological nodal (pN) stage, pathological tumor (pT) stage, pathological metastasis stage, chemotherapy, echinoderm microtubule-associated protein-like 4- anaplastic lymphoma kinase (EML4-ALK) fusion, and pathological type (**Figure 1A** and **Supplementary Figure 1**).

By analyzing the incidence of somatic mutation in 86 CRGs above, we found a relatively high mutation frequency in the LUAD cohort (**Figure 1D**). Of the 507 LUAD samples, 375 (74%) had mutations in the CRGs (**Figure 1D**). Among them, *PRKDC, RB1*, and *CDKN2A* were the top three genes with high mutation frequency, while 12 CRGs (*TMEM191B*, *PDHB*, *IDH2*, *CERT1*, *MPC1*, *CEPT1*, *CIAO2A*, *CCDC137*, *C1QBP*, *SGF29*, *GCLM*, and *NME3*) had no mutations (**Figure 1D**). We explored CNV in these 86 CRGs. Among them, *UBAP2L*, *CHTOP*, *EGLN1*, *AHR*, and *HMGCS1* had widespread CNV increases, while *CDKN2A*, *CNN1*, *FZR1*, *LDLR*, and *RPAP1* showed CNV decreases (**Figure 1B**). The locations of the CNV alteration in the CRGs on their respective chromosomes are shown in **Figure 1C**.

Interestingly, the expression levels of the top 10 CRGs were positively correlated with CNV alteration between primary tumor and normal tissues. CRGs with CNV loss, such as *LDLR*, *CEPT1*, and *CNN1*, were expressed at lower levels in LUAD tissues compared to those in normal tissues, while CRGs with CNV gains, such as *CHTOP* and *UBAP2L*, were significantly increased in LUAD samples (**Figures 1A, B**). These results showed that CNV might regulate CRG mRNA expression. However, some CRGs with CNV loss, such as *IDH2*, *MTHFD1L*, and *ABCE1*, showed upregulated mRNA expression, while other CRGs with CNV gain, such as *TPK1*, showed downregulated mRNA expression between tumor and normal samples. Therefore, CNV was not the only factor to regulate gene expression, although many of the observed changes in CRG expression might be explained by CNV alteration. Like some other factors, transcription factors and DNA methylation might also be involved in CRG regulation.

Our analysis revealed significant differences in the genetic landscape and expression levels of CRGs between LUAD and normal tissues, suggesting a potential role of CRGs in LUAD tumorigenesis.

### Development and validation of cuproptosis consensus clusters in LUAD

**Figure 2:**
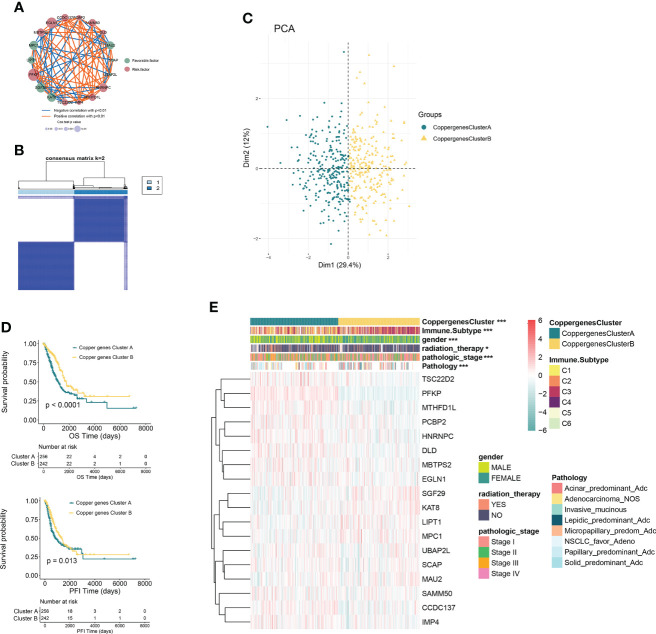
CRG subtypes and clinicopathological and biological characteristics of two distinct subtypes of samples divided by consistent clustering. **(A)** Interactions among CRGs in LUAD. The line connecting the CRGs represents their interaction, with the line thickness indicating the strength of the association between CRGs. Blue and orange represent negative and positive correlations, respectively. **(B)** Consensus matrix heatmap defining two clusters (k = 2) and their correlation area. **(C)** PCA analysis showing a remarkable difference in transcriptomes between the two subtypes. **(D)** Kaplan–Meier analysis showing 18 CRGs related to the PFI and OS (Copper genes Cluster A: 263; Copper genes Cluster B: 244). **(E)** Differences in clinicopathologic features and expression levels of CRGs between the two distinct subtypes. *P<0.05, ***P<0.001. CRGs, cuproptosis-related genes; LUAD, lung adenocarcinoma; TCGA, The Cancer Genome Atlas; PCA, principal components analysis; PFI, progression free interval; OS, overall survival.

In order to comprehensively understand the expression pattern of CRGs in the LUAD tumorigenesis, based on these 86 CRGs, a univariable Cox regression analysis was performed to screen the DEGs associated with a good prognosis. With the median DEGs expression values as the cutoff value, it was divided into high and low expression groups. The OS and PFI between high and low expression groups were compared by Kaplan-Meier analysis with the log-rank test, and *p*<0.05 was selected as the threshold for filtering. Finally, 18 CRGs with significant OS prognostic values were identified (**Supplementary Figure 2**). The comprehensive landscape of CRGs interactions, connection strength, and their prognostic value in LUAD patients was demonstrated in a cuproptosis network (**Figure 2A**).

In order to further explore the expression characteristics of CRGs in LUAD, a consensus clustering algorithm was used to categorize the individuals with LUAD according to the expression profiles of the 18 CRGs. Our results suggested that k = 2 appeared to be an optimal selection for dividing the entire cohort into subtypes A (n=263) and B (n=249) (**Figure 2B**). PCA analysis showed that there were significant differences in the cuproptosis transcription profiles between subtypes A and B (**Figure 2C**). Kaplan–Meier analysis revealed a longer OS and PFI in patients with subtype B than that in patients with subtype A (log-rank test, *p*<0.0001, *p*=0.013, respectively; **Figure 2D**). Furthermore, comparisons of the clinicopathological characteristics of the two subtypes of LUAD revealed significant differences between clinicopathological features and CRGs expression (**Figure 2E**).

### Characteristics of the TME between the two consensus clusters

**Figure 3:**
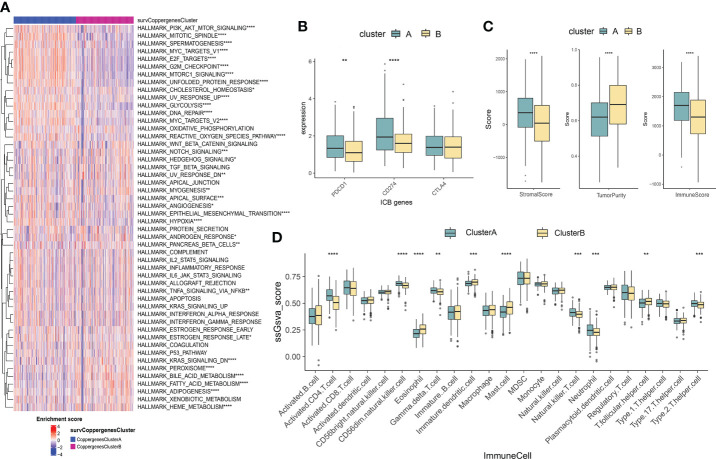
Characteristics of the TME between the two consensus clusters. **(A)** GSVA of biological pathways between two consensus clusters, in which red represent activated and blue represent inhibited pathways, respectively. **(B)** Expression levels of PDCD1 (PD-1), CD274 (PD-L1), and CTLA4 in the two consensus clusters. **(C)** Correlations between the two consensus clusters and TME score (stromal score, tumor purity, and immune score). *P<0.05, **P<0.01, ***P<0.001, **** P<0.0001. **(D)** Abundance of 23 infiltrating immune cell types in the two consensus clusters. GSVA, gene set variation analysis; TME, tumor microenvironment.

GSVA enrichment analysis showed that the signaling pathways with significant differences between the two groups were mainly enriched in the immune microenvironment signaling pathway, including *PI3K-AKT* signaling, *mTORC1* signaling, unfolded protein response, *MYC* target, *Notch* signaling, epithelial-mesenchymal transition, and *KRAS* signaling (**Figure 3A**). To investigate the role of CRGs in the TME of LUAD, we compared the difference in ssGSEA scores of 23 human immune cell subsets between the two clusters. We observed significant differences in the most immune cells infiltration levels between the two subtypes (**Figure 3D**, **Supplementary Figure 3**). The infiltration levels of activated CD4 T cell, CD56dim natural killer cell, gamma delta T cell, natural killer cell, neutrophil, type 2 T helper cell were obviously higher in the cluster A than those in the cluster B, while eosinophil, immature dendritic cell, mast cell, T follicular helper cell had significantly lower infiltration in cluster A compared to those in cluster B. We further analyzed two important immune checkpoints and found that the expression of PD1 and PD-L1 increased in cluster A (**Figure 3B**). We also evaluated the TME score using the ‘ESTIMATE’ package and found that immune and stromal scores were elevated in cluster A (**Figure 3C**), which suggested higher relative contents of immunocytes or stromal cells in the TME. In contrast, the tumor purity decreased significantly in cluster A. The results demonstrated higher TME levels in patients with cluster A.

### Identification of DEGs subtypes based on cuproptosis consensus clusters

**Figure 4:**
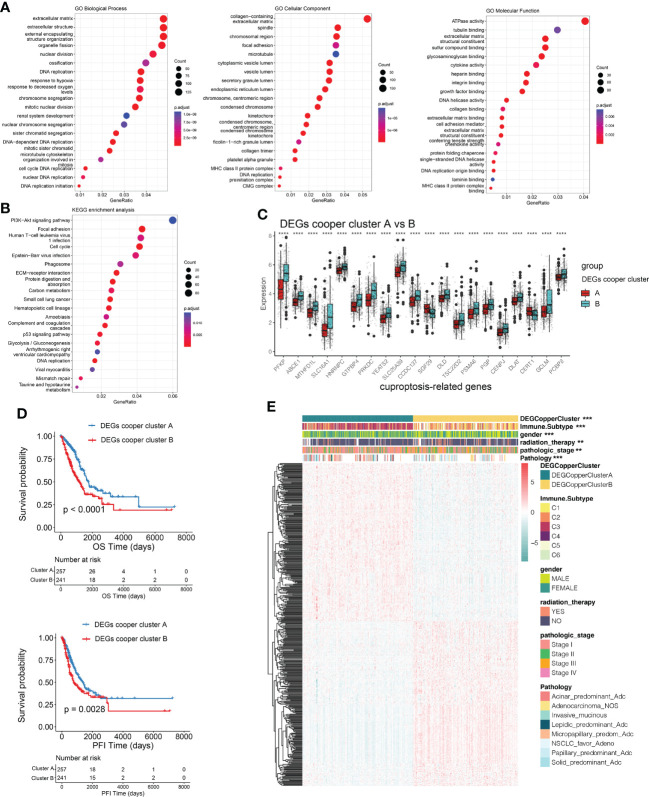
Identification of DEGs subtypes based on cuproptosis consensus clusters. **(A, B)** GO and KEGG enrichment analyses of DEGs among two cuproptosis consensus clusters. **(C)** Differences in the expression of CRGs among the two DEGs subtypes. **(D)** Kaplan–Meier curves for OS and PFI of the two DEGs subtypes (DEG Copper Cluster A: 260; DEG Copper Cluster B: 247) (log-rank tests, *p* <.001). **(E)** Relationships between clinicopathologic features and the two DEGs subtypes. **P<0.01, ***P<0.001, ****P<0.0001. DEGs, differentially expressed genes; GO, Gene Ontology; KEGG, Kyoto Encyclopedia of Genes and Genomes; CRGs, cuproptosis-related genes; PFI, progression free interval; OS, overall survival.

To explore the potential biological behavior of cuproptosis pattern, we identified 3153 DEGs between two cuproptosis consensus clusters and performed a functional enrichment analysis (**Figures 4A, B**). These CRGs were significantly enriched in biological processes that were correlated with extracellular matrix organization, extracellular structure organization, external encapsulating structure organization, organelle fission, nuclear fission, DNA replication, and chromosome segregation (**Figure 4A**). KEGG analysis indicated enrichment of *PI3K-Akt* signaling, focal adhesion, cell cycle, extracellular matrix receptor interaction, *p53* signaling pathway (**Figure 4B**), suggesting that cuproptosis plays a vital role in tumor genesis and progression.

We then conducted univariable Cox regression and Kaplan–Meier analysis, revealing the significant good prognostic values of 474 DEGs in patients with LUAD. In order to further validate the mechanism related to cuproptosis, we used a consensus clustering algorithm to divide LUAD patients into two gene subtypes (DEGs-cooper-cluster A and B) according to prognostic genes. The two cuproptosis gene subtypes showed significant differences in CRG expression (**Figure 4C**), consistent with the expected results of the cuproptosis patterns. Kaplan-Meier analysis revealed a longer OS and PFI in patients with subtype A than in patients with subtype B (log-rank test, *p*<0.0001, *p*=0.0028, respectively; **Figure 4D**). Furthermore, significant differences between clinicopathological features and CRGs expression were observed in DEGs-cooper-cluster A and B (**Figure 4E**).

### Development and validation of the prognostic CSS

**Figure 5:**
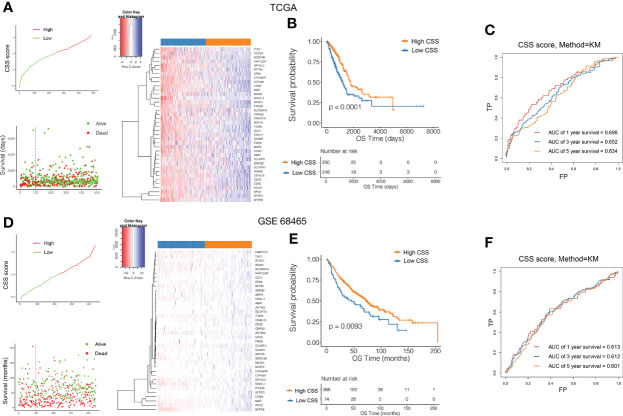
Validation of the prognostic value of CSS in the training and testing set. Ranked dot and scatter plots showing the CSS value distribution and patient survival status in TCGA **(A)** and GEO **(D)** database. Heatmaps of expression levels of prognostic DEGs in high and low CSS groups. Kaplan–Meier analysis of the OS between the high and low CSS groups in TCGA **(B)** and GEO **(E)** database. ROC curves to predict the sensitivity and specificity of 1-, 3-, and 5-year survival according to the CSS value in TCGA **(C)** and GEO **(F)** database. ROC, receiver operating characteristic; GO, Gene Ontology; KEGG, Kyoto Encyclopedia of Genes and Genomes; CSS, cuproptosis scoring system; DEGs, differentially expressed genes; OS, overall survival.

To quantify individual cuproptosis patterns in LUAD patients, we established a CSS based on the cuproptosis subtype-related DEGs. The CSS value was divided into high- and low- CSS groups. Using the TCGA as a training set and GEO (GSE11969, GSE72094, GSE68465) as a testing set to verify prognostic efficacy, we found that the prognosis of the high-CSS group was significantly better than that of the low-CSS group in both the data sets. The expression level of DEGs was higher in the high CSS group. The distribution plot of the risk of CSS value revealed that mortality rates decreased while survival times increased with an increase in CSS value. Heatmap demonstrated the expression of DEGs between the high- and low-CSS value groups (**Figures 5A–D**). The Kaplan-Meier survival curves revealed that patients with high CSS values had a significantly favorable overall survival compared to that patient with low CSS values (log-rank test, *p*<0.0001, *p*=0.0093, *p*=0.0028, *p*<0.0001, respectively; **Figures 5B–E**, **Supplementary Figure 4A, B**). In addition, the 1-, 3-, and 5-year survival rates of CSS values were represented by area under curve (AUC) values of 0.698, 0.652, and 0.634, respectively. Similar results were verified by the GEO database (**Figures 5C–F**). Correlation plots showed that the similarity of TCGA samples and GEO databases (GSE11969, GSE72094, GSE68465) in terms of gene expression for CRG genes (**Supplementary Figure 4C**).

**Figure 6:**
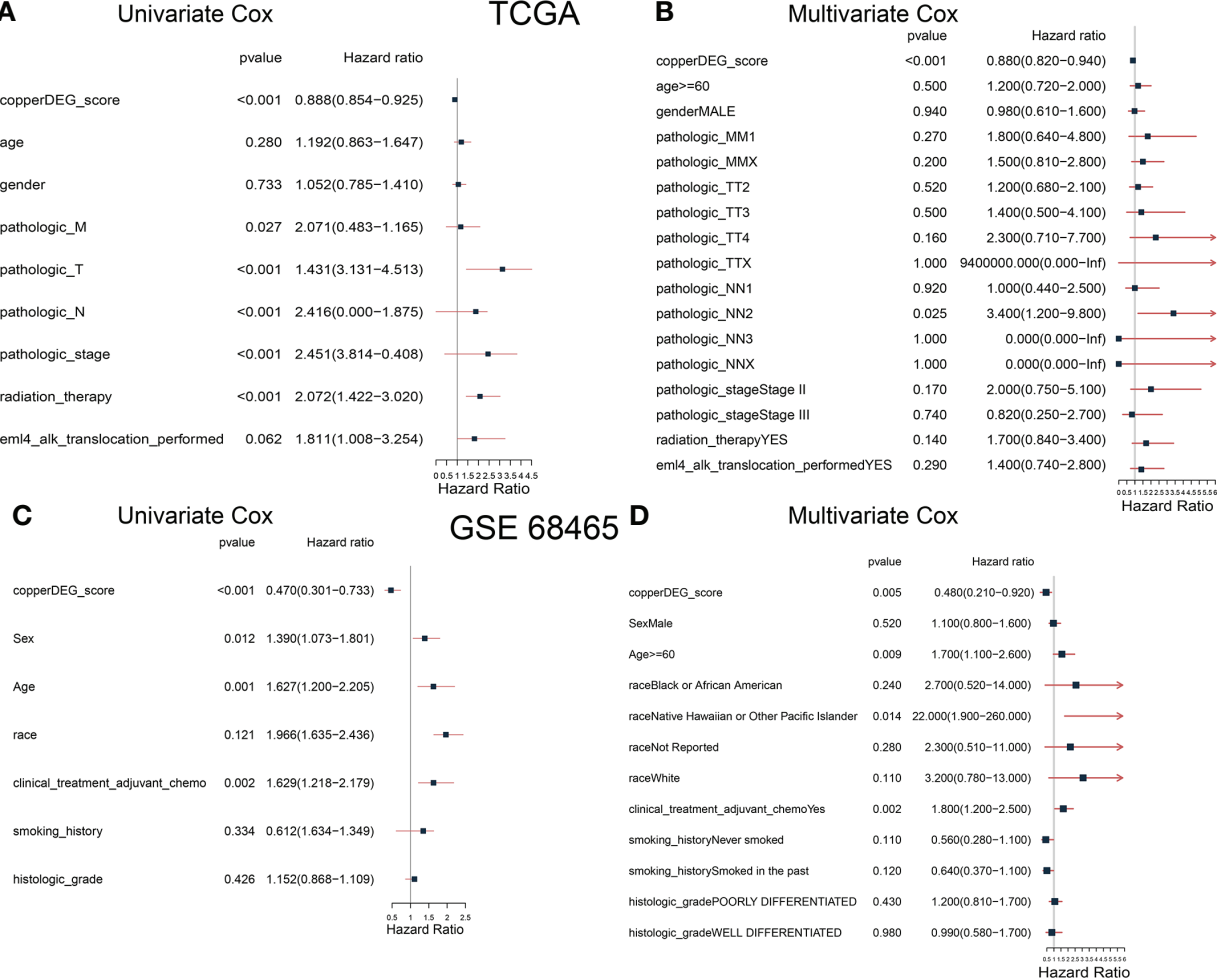
The correlation and independent prognosis analysis of CSS value and clinicopathological variables in LUAD. Univariable analyses showed the prognostic value of the CSS in the training **(A)** and testing **(C)** set. Multivariable analyses showed the prognostic value of the CSS in the training **(B)** and testing **(D)** set. CSS, cuproptosis scoring system; LUAD, lung adenocarcinoma.

To determine whether CSS value might independently predict OS in patients with LUAD, we combined the clinical features (age, sex, and TNM stage et al.) with CSS value to perform univariable and multivariable analyses. As shown in **Figures 6A, B**, the TNM stage and CSS value in the training set (TCGA) showed significant differences through univariable analyses, with consistent results observed in the testing set (**Figures 6C, D**). In multivariable analyses, CSS value was an independent factor that affects the OS in patients with LUAD in both training and testing sets.

**Figure 7:**
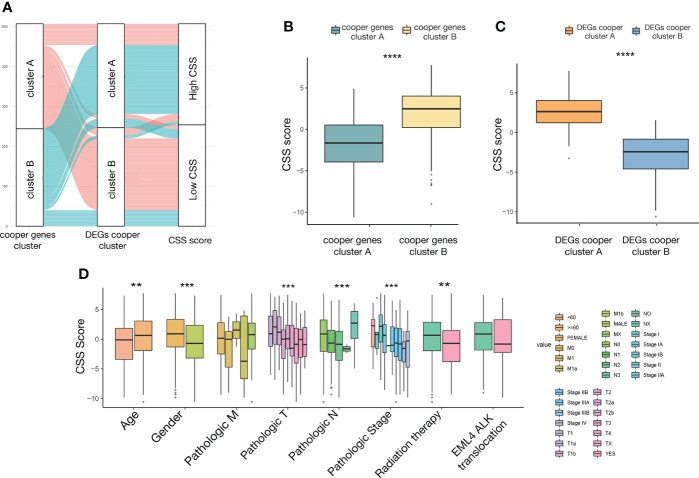
The relationship between CSS value and clinicopathological variables in LUAD. **(A)** alluvial diagram showing the distribution of patients in the two cuproptosis consensus clusters, two cuproptosis related DEGs subtypes, and two CSS score groups. **(B)** Differences in CSS value between two cuproptosis consensus clusters. **(C)** Differences in CSS value between two DEGs subtypes. **(D)** Differences in CSS value between different clinical characteristics. **P<0.01, ***P<0.001, ****P<0.0001. CSS, cuproptosis scoring system; LUAD, lung adenocarcinoma; DEGs, differentially expressed genes.

The distribution of patients in the two cuproptosis consensus clusters, two cuproptosis-related DEGs subtypes, and two CSS score groups was shown in **Figure 7A**. We observed a significant difference in CSS value between cuproptosis gene clusters. The CSS value of cluster A was lower than that of cluster B (**Figure 7B**). More importantly, compared to DEGs subtype B, DEGs subtype A had a significantly higher CSS value (**Figure 7C**).

To investigate the impact of the CSS value on clinical characteristics, we explored the relationship between CSS value and different clinical features, including age (≤60 and >60 years), gender (female and male), TNM stage (different T, N, M stage), pathological tumor stage (stage I, II, III, and IV), radiation therapy (yes and no), and EML4-ALK fusion (yes and no). We observed significantly higher CSS values in patients in age>60, female, relatively early T stage, N stage, pathological tumor stage, and did not receive radiation therapy subgroup relative to those in the corresponding subgroup (**Figure 7D**).

### Molecular characteristics of different CSS subgroups

**Figure 8:**
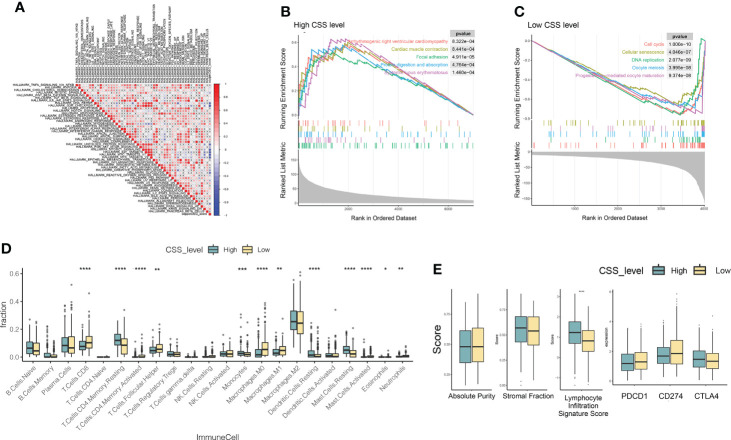
Molecular pathways of different CSS subgroups. **(A)** The Pearson correlation between CSS value and cancer hallmarks. **(B, C)** Gene sets enriched in CSS-high and CSS-low subgroup (*p* < 0.05, FDR < 0.25). **(D, E)** The distribution of immune cell subsets infiltration, absolute purity, stromal fraction, lymphocyte infiltration signature score, immune checkpoints between two CSS subgroups. *P<0.05, **P<0.01, ***P<0.001, ****P<0.0001. CSS, cuproptosis scoring system.

Based on MSigDB cancer hallmarks, the correlation between CSS value and cancer hallmarks was calculated by Pearson correlation analysis. We found that the CSS value was positively correlated with myogenesis, hedgehog signaling, *Kras* signaling, bile acid metabolism, coagulation, heme metabolism, *uv* response *dn* (**Figure 8A**), while CSS values were negatively correlated with DNA repair, *G2M* checkpoint, unfolded protein response, *PI3K-AKT* signaling, *MTORC1* signaling, *E2F* targets, *MYC* targets v1, *MYC* targets v2, oxidative phosphorylation, glycolysis, *uv* response *up* (**Figure 8A**; *P*< 0.05, FDR<0.25). GSEA was performed to determine the gene sets enriched in different CSS subgroups. The gene sets of the CSS-high samples were enriched in arrhythmogenic right ventricular cardiomyopathy, cardiac muscle contraction, focal adhesion, protein digestion and absorption, systemic lupus erythematosus. And the CSS-high samples were enriched in cell cycle, cellular senescence, DNA replication, oocyte meiosis, progesterone-mediated oocyte maturation (**Figures 8B, C**).

To investigate the role of CSS value in the TME of LUAD, we compared the difference in ssGSEA scores of human immune cell subsets between the high- and low- CSS groups. We observed significant differences in the most immune cells infiltration levels between the two groups (**Figure 8D**). The infiltration levels of Dendritic cells resting, Mast cell resting, Monocytes, T cells CD4 memory resting, Mast Cells, Dendritic Cells were obviously higher in the high CSS group than those in the low CSS group, while Macrophages M0, Macrophages M1, Mast cell activated, Neutrophils, T cells CD4 memory activated, T cells CD8, T Cells Follicular Helper, Neutrophils 1 had significantly lower infiltration in the high CSS group compared to those in low CSS group. We further analyzed TME scores and immune checkpoints and then found that the lymphocyte infiltration signature score increased in the high CSS subgroup (**Figures 8D, E**).

**Figure 9:**
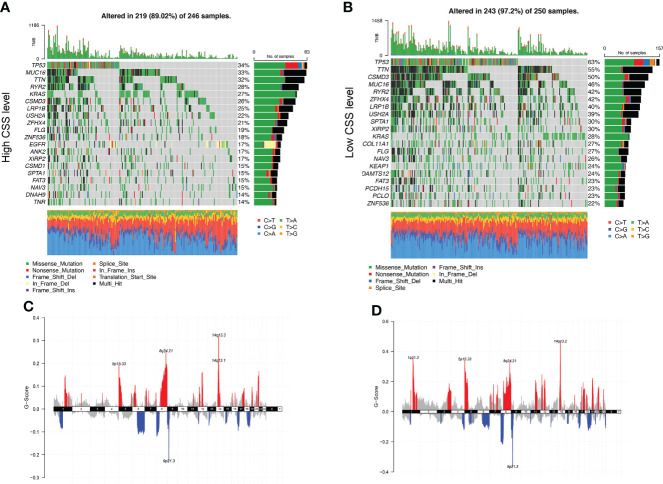
Genomic alteration of different CSS subgroups. **(A, B)** The waterfall plot of somatic mutation features established with high and low CSS subgroups. Each column represented an individual patient. The upper barplot showed TMB, the number on the right indicated the mutation frequency in each gene. The right barplot and color coding showed the proportion of each variant type. **(C, D)** Distribution of copy number amplification and deletion regions in high and low CSS subgroups. Red is amplification, and blue is deletion. CSS, cuproptosis scoring system; TMB, tumor mutation burden.

Next, we analyzed gene mutations to gain further biological insight in the CSS subgroups. We found significantly higher mutation counts in the low-CSS subgroup than in the high-CSS subgroup. Missense variations were the most common mutation type, followed by synonymous variations (**Figures 9A, B**). The mutation rates of *TP53, MUC16, TTN, RYR2, KRAS, CSMD3, LRP1B, USH2A, and ZFHX4* were higher than 20% in both groups. Compared with the low-CSS subgroup, the mutations of the *TP53*, *MUC16*, and *TTN* genes were more common in the high-CSS subgroup, while the mutation of *TP53*, *TTN*, and *CSMD3* genes were more common in the low-CSS subgroup than in high-CSS subgroup (**Figures 9A, B**). There was no significant difference in CNV between the two groups (**Figures 9C, D**, **Supplementary Figure 5**).

### The chemotherapeutic and targeted therapeutic sensitivity in different CSS subgroups

**Figure 10:**
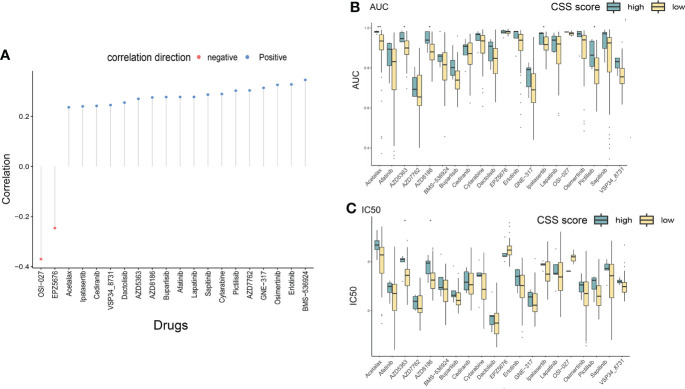
Drug susceptibility analysis of different CSS subgroups. **(A)** The correlation between CSS value and IC50/AUC of different chemotherapeutic and targeted drugs through Spearman’s method. **(B, C)** The differences in drug sensitivity (IC50/AUC) between high and low CSS groups were compared. *P<0.05, **P<0.01. IC50, 50% inhibiting concentrations; AUC, area under the curve; CSS, cuproptosis scoring system.

In order to improve the therapeutic benefit of LUAD patients from chemotherapy and targeted therapy, we further explored whether cuproptosis signature could predict the sensitivity to several drugs widely used in LUAD between two CSS groups. According to the results calculated based on the GDSC database, IC50 and AUC_IC50_ values of chemotherapy and targeted therapy drugs covering Acetalax, Cytarabine, EPZ5676, Pictilisib, GNE-317, AZD8186, Erlotinib, OSI-027, Buparlisib, Sapitinib, Ipatasertib, Dactolisib, AZD5363, Lapatinib, Osimertinib, Cediranib, VSP34 8731, Afatinib, AZD7762, and BMS-536924 were evaluated. The spearman correlation analysis showed that CSS values were positively correlated with IC50 and AUC_IC50_ values of most drugs, except for OSI-027 and EPZ5676 (**Figure 10A**). Compared with the high-CSS subgroup, IC50 values of AZD5363, and AZD8186 were lower in the low-CSS subgroup, which indicated that low-CSS patients were more sensitive to these drugs (**Figures 10B, C**). The above results demonstrated that the CSS value had potential predictive value for chemotherapy and targeted therapy in LUAD.

### The immune response in different CSS subgroups

**Figure 11:**
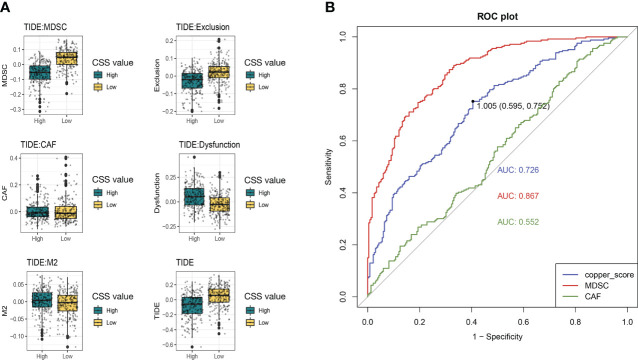
The immune response in different CSS subgroups. **(A)** The comparation of different forms of TIDE scores between high and low CSS subgroups. **(B)** ROC curves for predicting immune response. TIDE, tumor immune dysfunction and exclusion; ROC, receiver operating characteristic curve.

We then used the tumor immune dysfunction and exclusion (TIDE) score to assess the rise and fall of immune response in different CSS subgroups. A higher TIDE prediction score represented a higher potential for immune evasion, which suggested that the patients were less likely to benefit from immune checkpoint inhibitor (ICI) therapy. In our results, the high-CSS subgroup had a lower TIDE score than the low-CSS subgroup, implying that high-CSS patients could benefit more from ICI therapy than low-CSS patients (**Figure 11A**). Besides, a higher TIDE prediction score was associated with a worse outcome. Accordingly, the high-CSS subgroup with a low TIDE score might have a better prognosis than the low-CSS group with a high TIDE score.

In addition, we found that the low-CSS subgroup had a higher myeloid-derived suppressor cells (MDSC) and T-cell exclusion score, but there was no difference in T-cell dysfunction between the two subgroups. Moreover, we assessed the prediction of the TIDE immune response by CSS. Receiver operating characteristic (ROC) curve showed that the AUCs for CSS was 0.726, which was lower than MDSC (AUC=0.887) and higher than cancer associated fibroblasts (CAF) (AUC=0.552) (**Figure 11B**). We suggested that the CSS has great predictive value for immunotherapy.

## Discussion

Some studies described the unique significance of cuproptosis in predicting prognosis and explored the new target in other malignant tumors (12, 16). As we introduced previously, however, there was no related report on the association between cuproptosis and LUAD. Therefore, we performed the study to explore the key role of CRGs in the evolution of LUAD, identification of cuproptosis-related subtypes, characterization of TME, development of a prognostic model, and prediction of treatment response (including targeted and immune therapy). We first presented the landscape of cuproptosis-related gene mutations and CNV and screened out 18 genes affecting survival in LUAD. Based on those 18 CRGs, two molecular subtypes of cuproptosis were identified by K-means clustering. Patients with Copper Genes cluster B had better survival than those with Copper Genes cluster A. Besides, we further analyzed the immune regulation mechanism in different molecular subtypes of cuproptosis. We found that the score of ssGSEA to immune cell infiltration was different in the two clusters. For example, infiltration of activated CD4^+^ T cells, natural killer T cells, and neutrophils was stronger in cluster A than in cluster B. To validate the abovementioned molecular subtypes of cuproptosis, we calculated the DEGs according to those two clusters and reached 474 prognostic genes. Then, the K-nearest neighbor method was conducted to confirm the subtypes of DEGs and classify those 474 genes into two new clusters, DEG Copper cluster A and cluster B. The DEG Copper clusters also showed a good stratified effect on the prognosis of LUAD. In addition, the predictive index, CSS based on 474 genes, was developed and validated. The CSS was related to age, sex, radiotherapy, pT category, pN classification, *PDCD1*, *CTLA4*, and CD274. Besides, the subsequent analysis found that CSS was associated with multiple tumor hallmark pathways and immune cells. We also explored the significance of CSS in predicting the effect of the drug and uncovering novel targets. Finally, we found that high-level CSS might imply a better AUC value of drugs, including Dacomitinib, Osimertinib, and Erlotinib which were important to LUAD patients. In addition, CSS provided a better predictive ability to assess the effect of immune therapy, which had vital reference information about clinical practice.

Immunotherapy plays an important role in elevating the prognosis of LUAD patients. For patients with advanced and metastatic NSCLCs, ICIs were recommended as the first-line treatment (17). However, about 50% of NSCLC patients did not benefit from immunotherapy (31). We further investigated the correlation between the cuproptosis-related prognosis signature and TME, and the differences in TME characteristics and immunotherapy response between the two CSS subgroups, as TME was highly associated with immunotherapy response (32). The expression of immune checkpoints was highly distinct between two CSS groups. Specifically, the low CSS group had a significantly higher abundance of CD8+ T cells and CD4+ memory T cells than it in the high score CSS group. It is reported that tumor-infiltrating T cells, especially CD8+ T cells, were associated with tumor cell killing and response to ICIs (33). However, the high score CSS group had much improved outcomes than low score CSS group in the present study. This phenomenon may imply that the abundance of CD8+ T cells is not necessarily positively correlated with good prognosis; the reason may be that the number of CD8+ T cells in the TME had a weak association with the response to immunotherapy and patient prognosis. Besides, it is necessary to evaluate the ratio and abundance of immune, stromal, and tumor cell comprehensively. In this study, we calculated immune signature scores including the above and found that high CSS scores were positively correlated with high immune signature scores. Those results revealed that LUAD patients possibly might be more sensitive to immunotherapy in the group with high CSS values than those with low CSS scores. In addition, we compared the predictive ability of immune response among MDSC, CAF, and CSS, and we found that CSS had the potential possibility to predict the immune response. In the clinical practice, tumor mutation burden, PD-1, and PD-L1 are used as predictive tools for immunotherapy (31, 34, 35). However, those markers are limited by poor accuracy. Moreover, the test of PD-1, PD-L1, and MDSC were used in biopsy tissues routinely; however, CSS provided feasibility for blood testing to predict the immune response. Accordingly, CSS could be a predictive tool for immune therapy and prognostic assessment.

In the second cluster analysis based on 474 genes, we found that *PI3K-AKT-mTOR* signaling was the first ranked in the KEGG pathway analysis. *PI3K-AKT-mTOR* is downstream of *EGFR* and *Kras* (36). The resistance of Erlotinib was related to the *PI3K-AKT-mTOR* signaling according to reports (37). Thus, the CSS might be an approach to observe the resistance of some targeted drugs. In addition, AZD5363 and AZD8186 were the inhibitors of *AKT* and *PI3K*, respectively, and had lower IC50 and AUC in the low-score CSS group than it in the high-score CSS group. Those findings suggested that patients with the low-score CSS were more sensitive to targeted therapy than those with high-score CSS. According to our results, patients in the low-score CSS group had a poor prognosis; however, those patients in the low-score CSS group might benefit from treatment of AZD5363 and AZD8186. We still need further clinical trials to demonstrate those results.

This study has some limitations. First, the information on some important clinical variables including neoadjuvant chemotherapy, chemoradiotherapy, and surgery was unavailable in some datasets, which may influence the exploration of TME and cuproptosis state. Second, the association between cuproptosis and TME needs additional experimental verification. Third, the findings of our study are further needed to be validated in the data of our hospital. Fourth, the results of some drug susceptibility studies still need to be further validated and explored in cell lines and animals.

## Conclusions

In conclusion, CRGs are associated with the development, TME, and prognosis of LUAD. Besides, a scoring system based on CRGs can predict the efficacy of targeted drugs (such as osimertinib). In addition, CRGs are significantly enriched in the *PI3K* pathway, and the drug sensitivity of AZD363 and AZD8186, the targeted inhibitors of this pathway, are different, so a scoring system based on CRGs may be able to guide medication. Finally, this scoring system is a potential predictor of the immune response.

## Data availability statement

The datasets presented in this study can be found in online repositories. The names of the repository/repositories and accession number(s) can be found in the article/**Supplementary Material**.

## Ethics statement

The studies involving human participants were reviewed and approved by Ethics committee of Shanghai Pulmonary Hospital. The ethics committee waived the requirement of written informed consent for participation.

## Author contributions

DX, CC, KL, L-LW, C-WL, and HC contributed to the study design, data collection, data analyses, data interpretation, and manuscript drafting. L-LW, H-MZ, and Z-YL contributed to data analyses and manuscript review. DX, KL, L-LW, C-WL, J-YQ and HC contributed to data interpretation and manuscript review. All authors contributed to the article and approved the submitted version.

## Funding

This research was funded by the Shanghai ShenKang Hospital Development Centre, grant number SHDC22020218; Science and Technology Commission of Shanghai Municipality, grant number 21Y11913400; Shanghai Pulmonary Hospital Foundation, grant number fkxr1904 & fkzr2131; National Natural Science Foundation of China, grant number 82200999 & 82272943; and Outstanding Young Medical Talent of Rising Star in Medical Garden of Shanghai Municipal Health Commission “Dong Xie”, grant number “Dong Xie”.

## Conflict of interest

The authors declare that the research was conducted in the absence of any commercial or financial relationships that could be construed as a potential conflict of interest.

## Publisher’s note

All claims expressed in this article are solely those of the authors and do not necessarily represent those of their affiliated organizations, or those of the publisher, the editors and the reviewers. Any product that may be evaluated in this article, or claim that may be made by its manufacturer, is not guaranteed or endorsed by the publisher.

## Abbreviation

LUAD, lung adenocarcinoma; TME, tumor microenvironment; CRGs, cuproptosis-related genes; TCGA, the cancer genome atlas; GEO, gene expression omnibus; FPKM, fragments per kilobase million; TPM, transcripts per kilobase million; GSVA, Gene set variation analysis; DEGs, differentially expressed genes; CNV, copy number variations; OS, overall survival; PFI, progression free interval; GO, gene ontology; KEGG, Kyoto Encyclopedia of Genes and Genomes; FDR, false discovery rate; CSS, cuproptosis scoring system; PCA, principal component analysis; ssGSEA, Single sample Gene Set Enrichment Analysis; EGFR, epidermal growth factor receptor; pN, pathological nodal; pT, pathological tumor; EML4-ALK, echinoderm microtubule-associated protein-like 4- anaplastic lymphoma kinase; AUC, area under curve; TIDE, tumor immune dysfunction and exclusion; ICI, immune checkpoint inhibitor; MDSC, myeloid-derived suppressor cells; CAF, cancer associated fibroblasts.

## References

[1] Siegel RL Miller KD Fuchs HE Jemal A. Cancer statistics, 2021. CA Cancer J Clin (2021) 71(1):7–33. doi: 10.3322/caac.21654 33433946

[2] Wu L-L Li C-W Li K Qiu L-H Xu S-Q Lin W-K. The difference and significance of parietal pleura invasion and rib invasion in pathological T classification with non-small cell lung cancer. Front Oncol (2022) 12. doi: 10.3389/fonc.2022.878482 PMC9096107 35574398

[3] Klarenbeek SE Aarts MJ van den Heuvel MM Prokop M Tummers M Schuurbiers OCJ. Impact of time-to-treatment on survival for advanced non-small cell lung cancer patients in the Netherlands: a nationwide observational cohort study. Thorax (2022) thoraxjnl-2021-218059. doi: 10.1136/thoraxjnl-2021-218059 35450944

[4] Silva V Abrão F Peres S Rosamilia G Hanriot R Abreu I. Prognostic factors in curative intent stage I lung adenocarcinoma: a retrospective hospital-based cohort study. Lancet Oncol (2022) 23 Suppl 1:S31. doi: 10.1016/S1470-2045(22)00430-2 35848402

[5] Wu LL Li CW Lin WK Qiu LH Xie D. Incidence and survival analyses for occult lung cancer between 2004 and 2015: a population-based study. BMC Cancer (2021) 21(1):1009. doi: 10.1186/s12885-021-08741-4 34496775 PMC8427887

[6] Goldstraw P Chansky K Crowley J Rami-Porta R Asamura H Eberhardt WE. The IASLC lung cancer staging project: Proposals for revision of the TNM stage groupings in the forthcoming (Eighth) edition of the TNM classification for lung cancer. J Thorac Oncol (2016) 11(1):39–51. doi: 10.1016/j.jtho.2015.09.009 26762738

[7] Wu YL Tsuboi M He J John T Grohe C Majem M. : Osimertinib in resected EGFR-mutated non-small-cell lung cancer. N Engl J Med (2020) 383(18):1711–23. doi: 10.1056/NEJMoa2027071 32955177

[8] Zheng X Lu T Wu S Peng W Miao Q Jiang K. Tumour response heterogeneity as a powerful independent predictor of treatment outcome in advanced lung adenocarcinoma: a retrospective analysis. Lancet Oncol (2022) 23 Suppl 1:S13. doi: 10.1016/S1470-2045(22)00412-0

[9] Wu L-L Liu X Jiang W-M Huang W Lin P Long H. Stratification of patients with stage IB NSCLC based on the 8th edition of the American joint committee on cancer (AJCC) staging manual. Front Oncol (2020) 10:571. doi: 10.3389/fonc.2020.00571 32373536 PMC7186345

[10] Daemen A Cooper JE Myrta S Wongchenko MJ Lin E Long JE. Transcriptional subtypes resolve tumor heterogeneity and identify vulnerabilities to MEK inhibition in lung adenocarcinoma. Clin Cancer Res (2021) 27(4):1162–73. doi: 10.1158/1078-0432.CCR-20-1835 33023953

[11] Wan R Bai L Cai C Ya W Jiang J Hu C. Discovery of tumor immune infiltration-related snoRNAs for predicting tumor immune microenvironment status and prognosis in lung adenocarcinoma. Comput Struct Biotechnol J (2021) 19:6386–99. doi: 10.1016/j.csbj.2021.11.032 PMC8649667 34938414

[12] Pan S Meng H Fan T Hao B Song C Li D. Comprehensive analysis of programmed cell death signature in the prognosis, tumor microenvironment and drug sensitivity in lung adenocarcinoma. Front Genet (2022) 13:900159. doi: 10.3389/fgene.2022.900159 35664309 PMC9157820

[13] Tsvetkov P Coy S Petrova B Dreishpoon M Verma A Abdusamad M. Copper induces cell death by targeting lipoylated TCA cycle proteins. Sci (New York NY) (2022) 375(6586):1254–61. doi: 10.1126/science.abf0529 PMC9273333 35298263

[14] Kahlson MA Dixon SJ. Copper-induced cell death. Sci (New York NY) (2022) 375(6586):1231–2. doi: 10.1126/science.abo3959 35298241

[15] Lv H Liu X Zeng X Liu Y Zhang C Zhang Q. Comprehensive analysis of cuproptosis-related genes in immune infiltration and prognosis in melanoma. Front Pharmacol (2022) 13:930041. doi: 10.3389/fphar.2022.930041 35837286 PMC9273972

[16] Bian Z Fan R Xie L. A novel cuproptosis-related prognostic gene signature and validation of differential expression in clear cell renal cell carcinoma. Genes (Basel) (2022) 13(5):851. doi: 10.3390/genes13050851 35627236 PMC9141858

[17] National Comprehensive Cancer Network. Non-small cell lung cancer (Version 3.2022). Available at: https://www.nccn.org/professionals/physician_gls/pdf/nscl.pdf (Accessed 16 March 2022).

[18] Forde PM Chaft JE Smith KN Anagnostou V Cottrell TR Hellmann MD. Neoadjuvant PD-1 blockade in resectable lung cancer. New Engl J Med (2018) 378(21):1976–86. doi: 10.1056/NEJMoa1716078 PMC6223617 29658848

[19] Colaprico A Silva TC Olsen C Garofano L Cava C Garolini D. TCGAbiolinks: an R/Bioconductor package for integrative analysis of TCGA data. Nucleic Acids Res (2016) 44(8):e71. doi: 10.1093/nar/gkv1507 26704973 PMC4856967

[20] Li B Ruotti V Stewart RM Thomson JA Dewey CN. RNA-Seq gene expression estimation with read mapping uncertainty. Bioinf (Oxford England) (2010) 26(4):493–500. doi: 10.1093/bioinformatics/btp692 PMC2820677 20022975

[21] Shedden K Taylor JM Enkemann SA Tsao MS Yeatman TJ Gerald WL. Gene expression-based survival prediction in lung adenocarcinoma: a multi-site, blinded validation study. Nat Med (2008) 14(8):822–7. doi: 10.1038/nm.1790 PMC2667337 18641660

[22] Edgar R Domrachev M Lash AE. Gene expression omnibus: NCBI gene expression and hybridization array data repository. Nucleic Acids Res (2002) 30(1):207–10. doi: 10.1093/nar/30.1.207 PMC99122 11752295

[23] Irizarry RA Bolstad BM Collin F Cope LM Hobbs B Speed TP. Summaries of affymetrix GeneChip probe level data. Nucleic Acids Res (2003) 31(4):e15. doi: 10.1093/nar/gng015 12582260 PMC150247

[24] Ritchie ME Phipson B Wu D Hu Y Law CW Shi W. Limma powers differential expression analyses for RNA-sequencing and microarray studies. Nucleic Acids Res (2015) 43(7):e47. doi: 10.1093/nar/gkv007 25605792 PMC4402510

[25] Yu G Wang LG Han Y He QY. clusterProfiler: an r package for comparing biological themes among gene clusters. Omics J Integr Biol (2012) 16(5):284–7. doi: 10.1089/omi.2011.0118 PMC3339379 22455463

[26] Hänzelmann S Castelo R Guinney J. GSVA: gene set variation analysis for microarray and RNA-seq data. BMC Bioinf (2013) 14:7. doi: 10.1186/1471-2105-14-7 PMC3618321 23323831

[27] Liberzon A Birger C Thorvaldsdóttir H Ghandi M Mesirov JP Tamayo P. The molecular signatures database (MSigDB) hallmark gene set collection. Cell Syst (2015) 1(6):417–25. doi: 10.1016/j.cels.2015.12.004 PMC4707969 26771021

[28] Mayakonda A Lin DC Assenov Y Plass C Koeffler HP. Maftools: efficient and comprehensive analysis of somatic variants in cancer. Genome Res (2018) 28(11):1747–56. doi: 10.1101/gr.239244.118 PMC6211645 30341162

[29] Newman AM Liu CL Green MR Gentles AJ Feng W Xu Y. Robust enumeration of cell subsets from tissue expression profiles. Nat Methods (2015) 12(5):453–7. doi: 10.1038/nmeth.3337 PMC4739640 25822800

[30] Thorsson V Gibbs DL Brown SD Wolf D Bortone DS Ou Yang TH. The immune landscape of cancer. Immunity (2018) 48(4):812–30.e14. doi: 10.1016/j.immuni.2018.03.023 29628290 PMC5982584

[31] Gadgeel S Rodríguez-Abreu D Speranza G Esteban E Felip E Dómine M. Updated analysis from KEYNOTE-189: Pembrolizumab or placebo plus pemetrexed and platinum for previously untreated metastatic nonsquamous non-Small-Cell lung cancer. J Clin Oncol (2020) 38(14):1505–17. doi: 10.1200/JCO.19.03136 32150489

[32] Huang CP Liu LC Chang CC Wu CC Shyr CR. Intratumoral xenogeneic tissue-specific cell immunotherapy inhibits tumor growth by increasing antitumor immunity in murine triple negative breast and pancreatic tumor models. Cancer Lett (2022) 545:115478. doi: 10.1016/j.canlet.2021.10.044 35902043

[33] Nguyen TT Shin DH Sohoni S Singh SK Rivera-Molina Y Jiang H. Reshaping the tumor microenvironment with oncolytic viruses, positive regulation of the immune synapse, and blockade of the immunosuppressive oncometabolic circuitry. J Immunother Cancer (2022) 10(7):e004935. doi: 10.1136/jitc-2022-004935 35902132 PMC9341188

[34] Felip E Altorki N Zhou C Csőszi T Vynnychenko I Goloborodko O. Adjuvant atezolizumab after adjuvant chemotherapy in resected stage IB-IIIA non-small-cell lung cancer (IMpower010): a randomised, multicentre, open-label, phase 3 trial. Lancet (London England) (2021) 398(10308):1344–57. doi: 10.1016/S0140-6736(21)02098-5 34555333

[35] Rizvi H Sanchez-Vega F La K Chatila W Jonsson P Halpenny D. Molecular determinants of response to anti-programmed cell death (PD)-1 and anti-programmed death-ligand 1 (PD-L1) blockade in patients with non-Small-Cell lung cancer profiled with targeted next-generation sequencing. J Clin Oncol (2018) 36(7):633–41. doi: 10.1200/JCO.2017.75.3384 PMC6075848 29337640

[36] Burns TF Borghaei H Ramalingam SS Mok TS Peters S. Targeting KRAS-mutant non-small-cell lung cancer: One mutation at a time, with a focus on KRAS G12C mutations. J Clin Oncol (2020) 38(35):4208–18. doi: 10.1200/JCO.20.00744 PMC7723684 33104438

[37] Abdel-Wahab R Varadhachary GR Bhosale PR Wang X Fogelman DR Shroff RT. Randomized, phase I/II study of gemcitabine plus IGF-1R antagonist (MK-0646) versus gemcitabine plus erlotinib with and without MK-0646 for advanced pancreatic adenocarcinoma. J Hematol Oncol (2018) 11(1):71. doi: 10.1186/s13045-018-0616-2 29843755 PMC5975422

