# Supplementary figures and images for: The characterization of tumor microenvironment infiltration and the construction of predictive index based on cuproptosis-related gene in primary lung adenocarcinoma
Source: Front Oncol. 2022 Nov 25;12:1011568. doi: 10.3389/fonc.2022.1011568 (PMC9733577; doi:10.3389/fonc.2022.1011568)

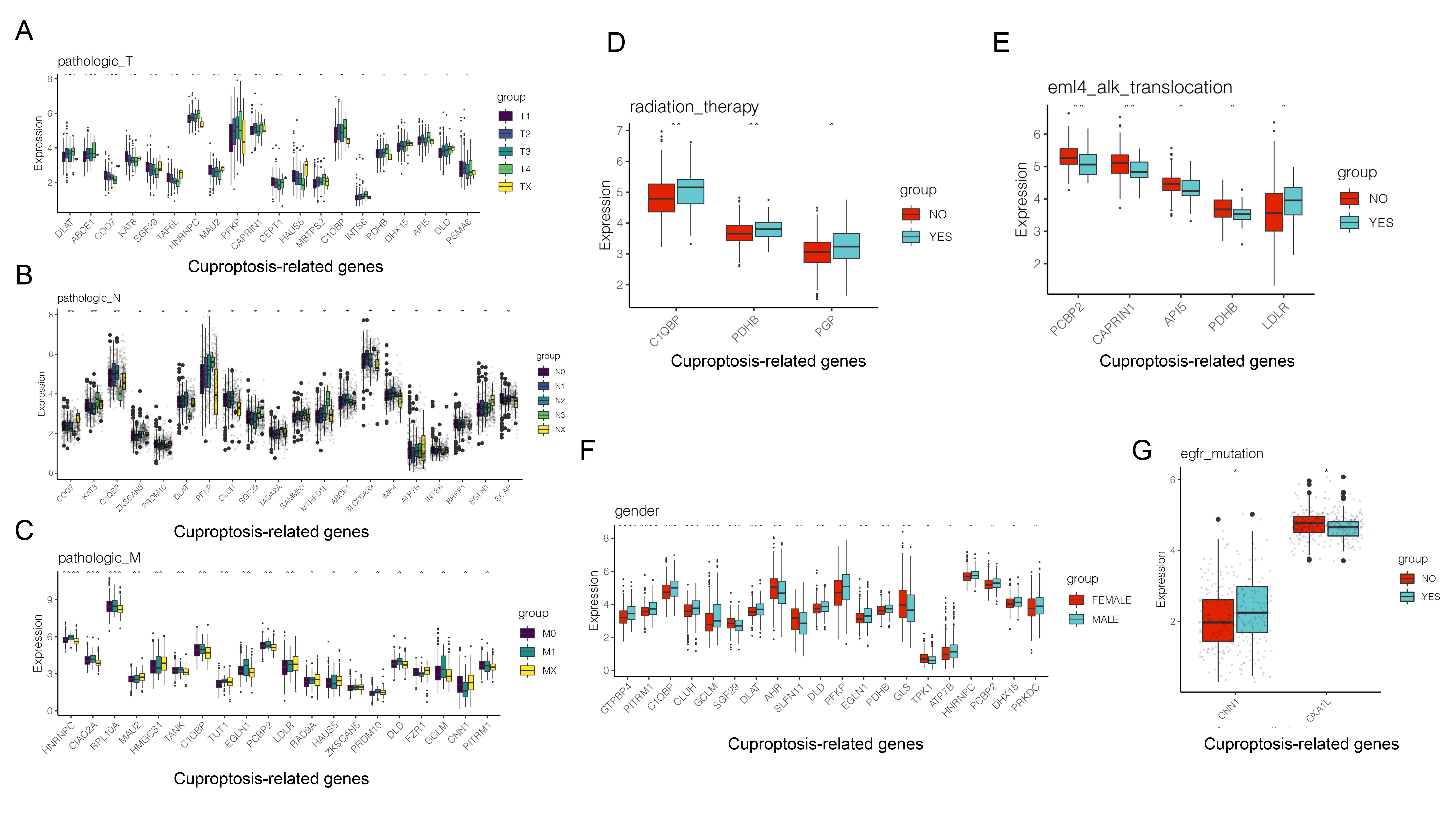

Supplement: Supplementary file 1 [file Image_1.tif]

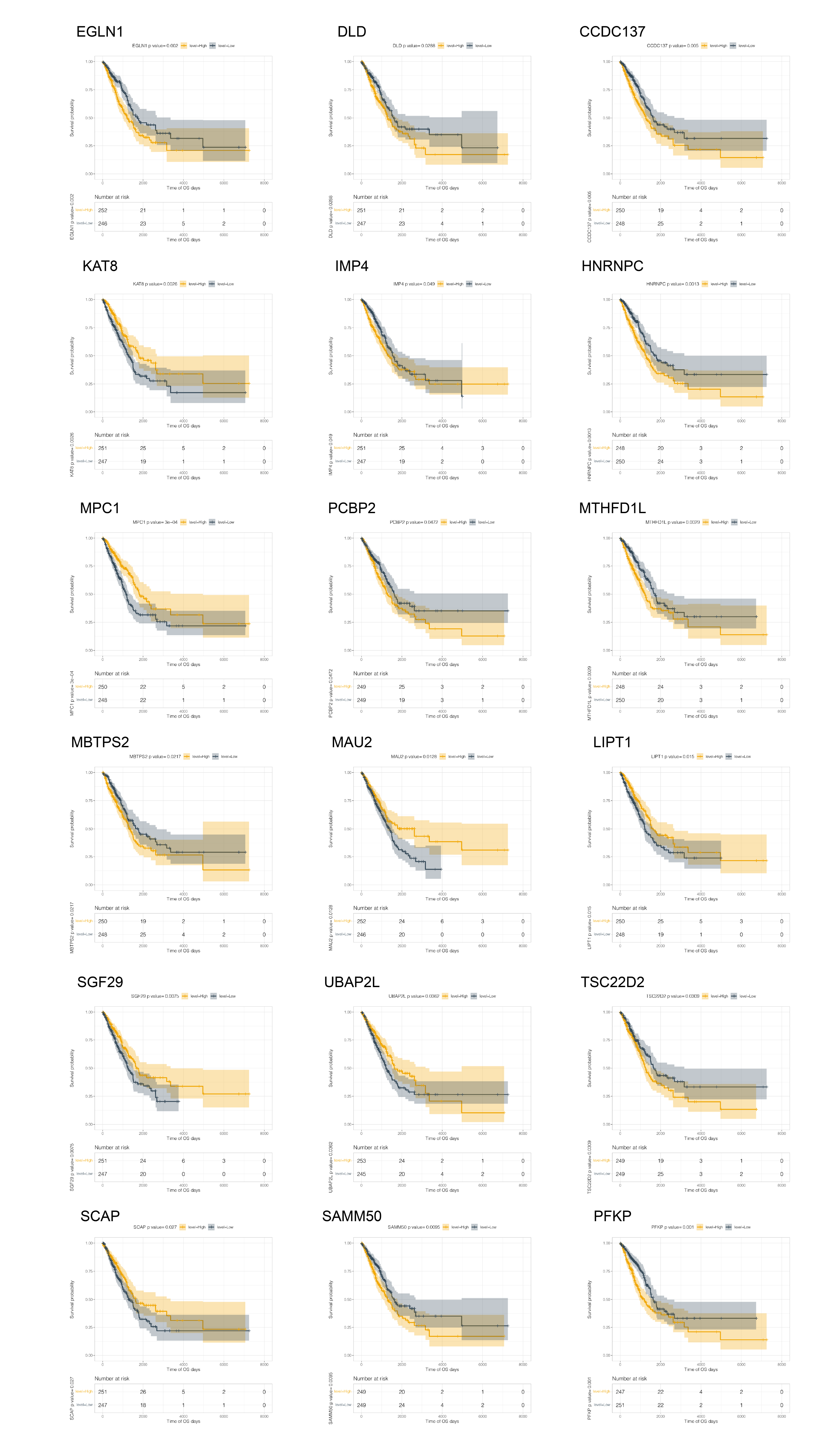

Supplement: Supplementary file 2 [file Image_2.tif]

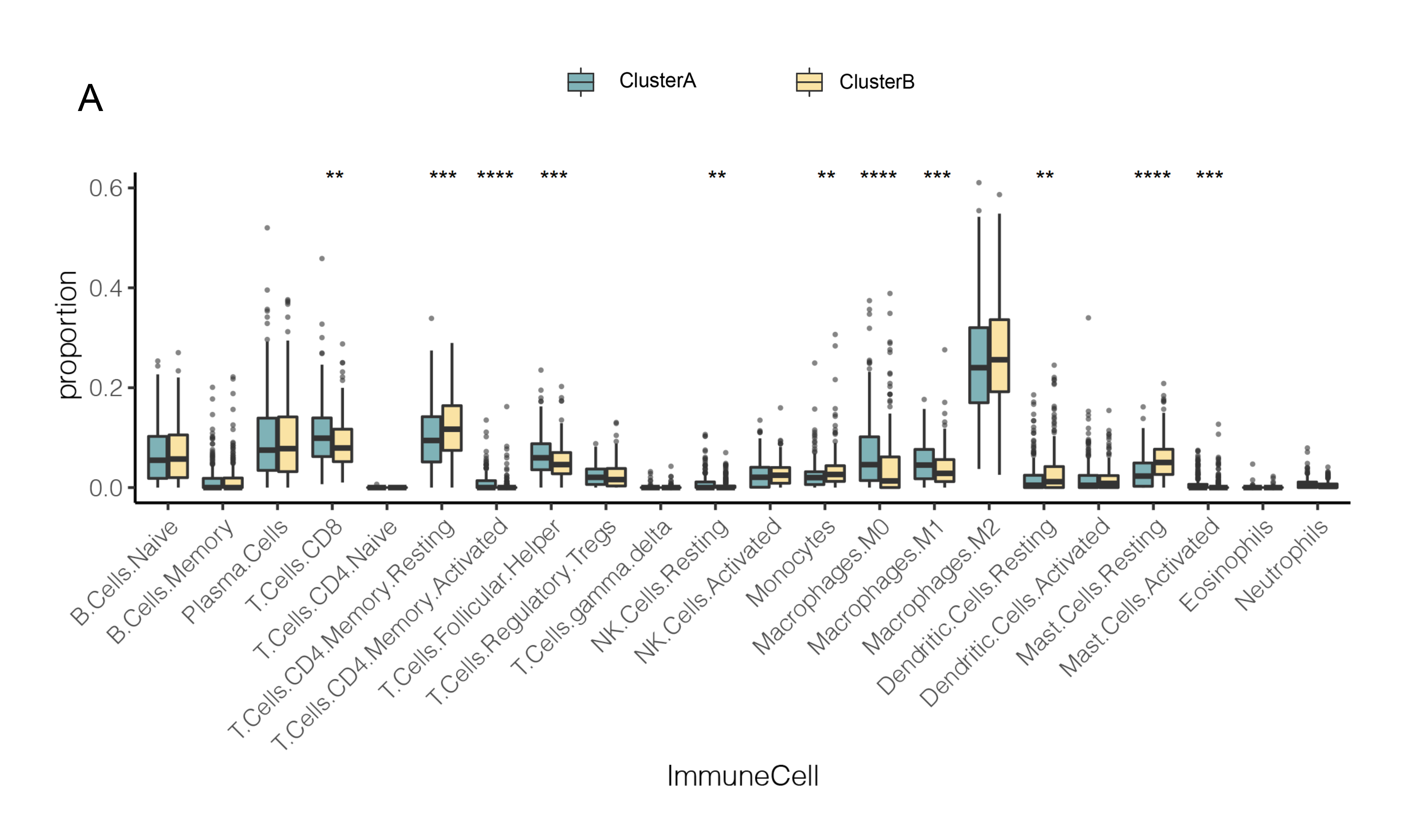

Supplement: Supplementary file 3 [file Image_3.tif]

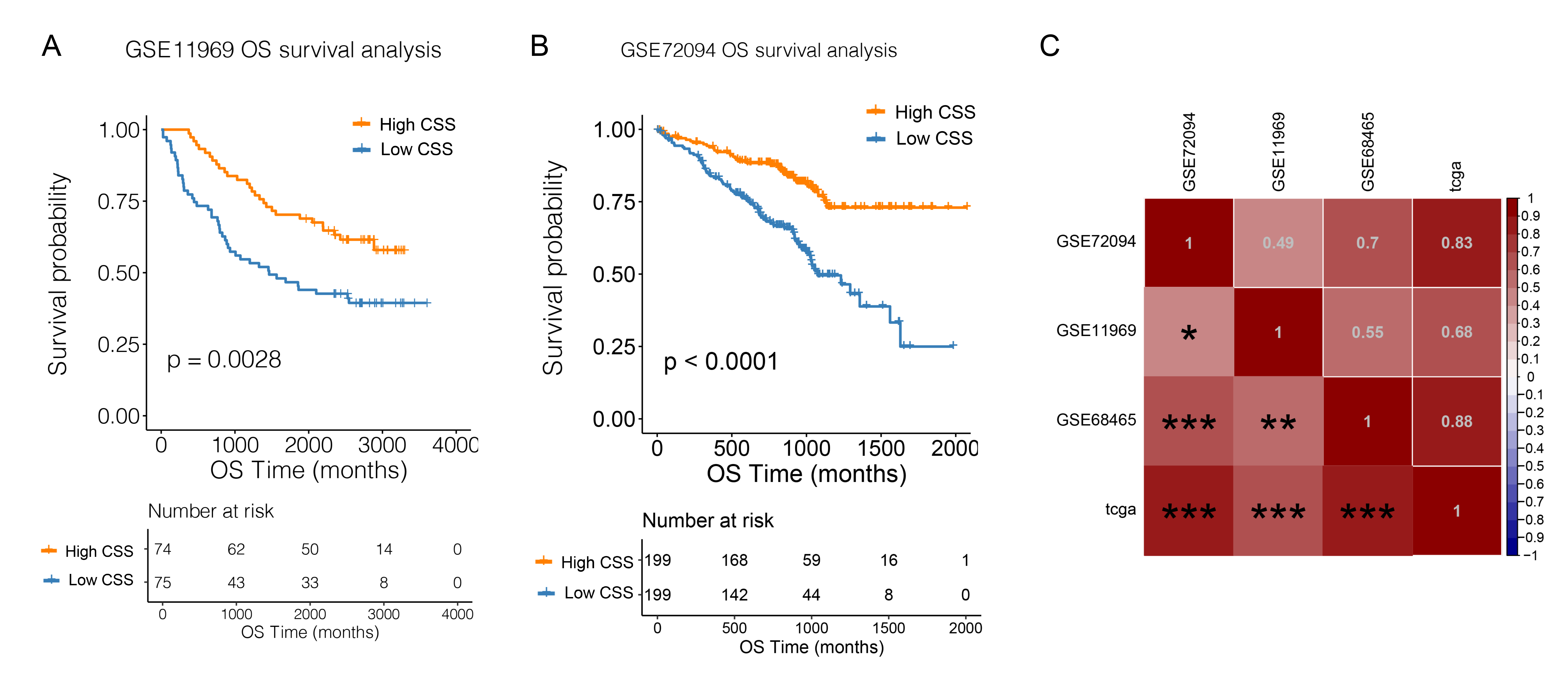

Supplement: Supplementary file 4 [file Image_4.tif]

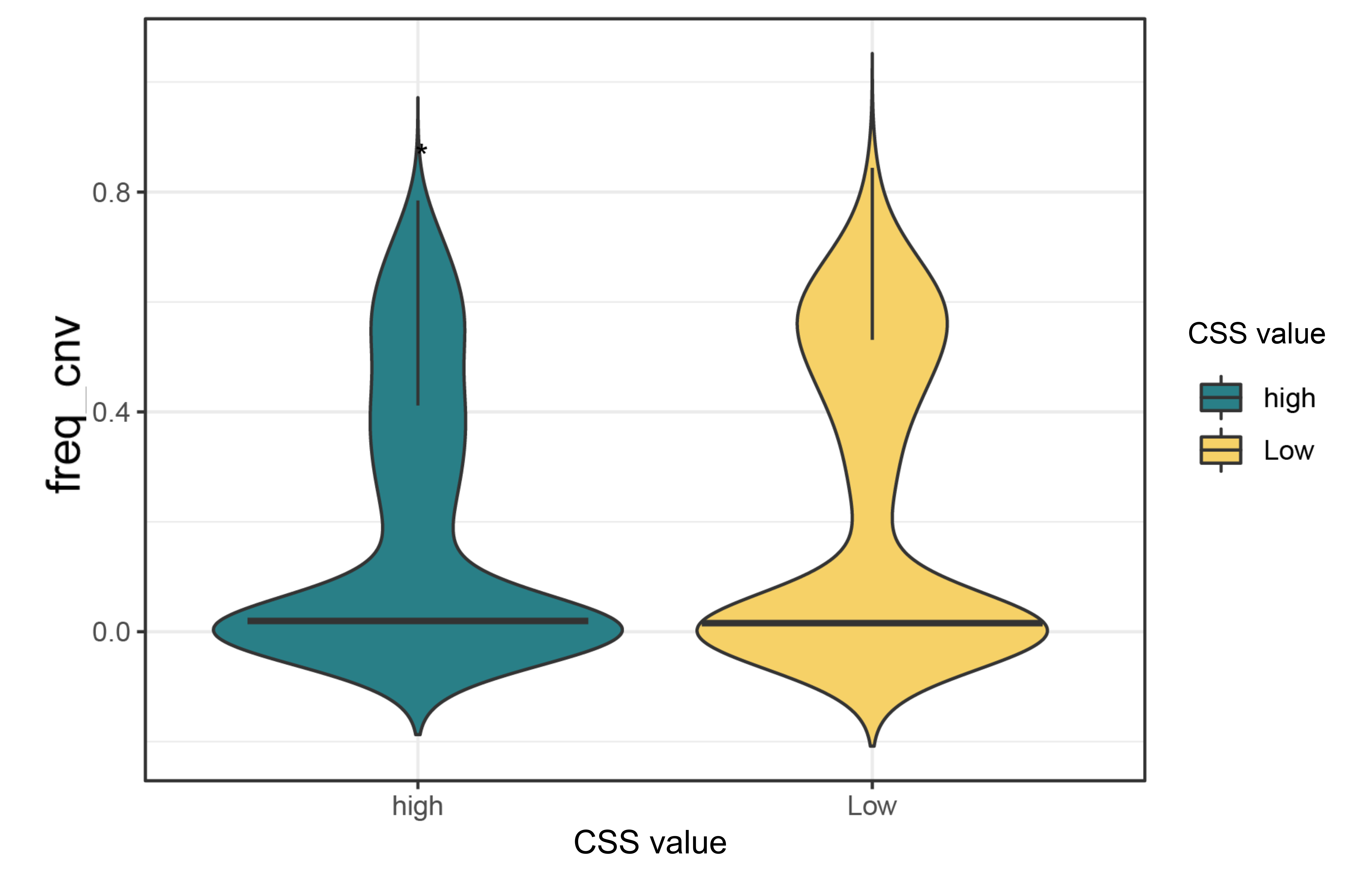

Supplement: Supplementary file 5 [file Image_5.tif]
