# Supplementary material for: The characterization of tumor microenvironment infiltration and the construction of predictive index based on cuproptosis-related gene in primary lung adenocarcinoma
Source: Front Oncol. 2022 Nov 25;12:1011568. doi: 10.3389/fonc.2022.1011568 (PMC9733577; doi:10.3389/fonc.2022.1011568)
Supplement: Supplementary file 7 [file DataSheet_1.docx]

**SUPPLEMENTARY TABLE**

The 100 CRGs were obtained from Tsvetkov’s study

**SUPPLEMENTARY FIGURE 1**

Expression levels of CRGs in LUAD. (A–G) Expression of CRGs in clinical Q28 samples classified by pathological nodal (pN) stage, pathological tumor (pT) stage, pathological metastasis stage, epidermal growth factor

receptor (EGFR) mutation, echinoderm microtubule-associated proteinlike 4-anaplastic lymphoma kinase (EML4-ALK) fusion, gender and radiation therapy.

**SUPPLEMENTARY FIGURE 2**

18 CRGs with significant OS prognostic values were identified.

**SUPPLEMENTARY FIGURE 3**

Profiling tumor infiltrating immune cells with CIBERSORT in the two consensus clusters.

**SUPPLEMENTARY FIGURE 4**

Kaplan–Meier analysis of the OS between the high and low CSS groups in GSE11969 (A) and GSE72094 (B) database. Pearson correlation was used to calculate the correlation of CRGs expression among TCGA and three

GEO database (GSE68465, GSE11969, GSE72094 data sets).

**SUPPLEMENTARY FIGURE 5**

The CNV alteration of the CRGs in high and low CSS subgroups.
